# Reduced Nitrogen Application with Dense Planting Achieves High Eating Quality and Stable Yield of Rice

**DOI:** 10.3390/foods13183017

**Published:** 2024-09-23

**Authors:** Yajie Hu, Liang Sun, Jiantao Xue, Qin Cai, Yi Xu, Jinghao Guo, Haiyan Wei, Zhongyang Huo, Ke Xu, Hongcheng Zhang

**Affiliations:** 1 Jiangsu Key Laboratory of Crop Cultivation and Physiology, Agricultural College of Yangzhou University, Yangzhou 225009, China; huyajie@yzu.edu.cn (Y.H.);; 2 Co-Innovation Center for Modern Production Technology of Grain Crops, Yangzhou University, Yangzhou 225009, China

**Keywords:** rice, reduced nitrogen, dense planting, grain yield, eating quality, starch

## Abstract

Rational nitrogen (N) application can enhance yield and improve grain eating quality in rice. However, excessive N input can deteriorate grain eating quality and aggravate environmental pollution, while reduced N application (RN) decreases rice yield. Reduced N application with dense planting (RNDP) is recommended for maintaining rice yield and improving N use efficiency. However, the effects of RNDP on the rice grain eating quality and starch structure and properties remain unclear. A two-year field experiment was conducted to investigate the effects of RNDP on the rice yield, grain eating quality, and starch structure and properties. Compared to conventional N treatment, RN decreased significantly the rice yield, while RNDP achieved a comparable grain yield. Both the RN and RNDP treatments improved significantly the rice eating quality. The high eating quality of RNDP was attributed to increased gel consistency, pasting viscosity, and stickiness after cooking as well as decreased protein content. A further analysis of starch structure and properties revealed that RNDP decreased the relative crystallinity, lamellar intensity, gelatinization enthalpy, and retrogradation enthalpy of starch. Therefore, RNDP achieved a stable rice yield and enhanced rice eating quality. These findings provide valuable insights into obtaining optimal quality and consistent yield in rice production under reduced N conditions.

## 1. Introduction

Rice (*Oryza sativa* L.) is the world’s most important grain crop, especially in Asia, and it serves as a primary food for over 50% of the global population, providing 20% of the world population’s nutritional and calorie requirements [1]. In China, rice is the staple cereal food for over 65% of the population [2]. In recent decades, the overall rice output has remained stable at more than 200 million tons in China, and the rice grain yield per unit area has increased continuously with fluctuations, owing to the innovations of rice variety breeding and cultivation technology. With the increasing improvement in living standards, the high taste quality of rice is emphasized and preferred by consumers. Therefore, the focus of rice production and consumptive demand has shifted to quality over quantity [3]. The question of how to acquire rice with high-yield and high-taste quality is a particularly great challenge in addition to achieving rice production with fewer resource inputs and lower environmental costs. 

Rice quality is an increasingly popular topic for breeders and agronomists, especially rice eating and cooking quality, which is considered by many consumers to be a quality trait of primary importance. Rice eating and cooking quality is a multifaceted trait related to the starch, proteins, and lipids in kernels [4,5]. Rice grains consist of 80–85% starch, 6–10% proteins, and 1–3% lipids. The starch components and fine structure, protein content, and gel consistency further impact the eating and cooking quality of rice [6]. Previous studies have shown that rice with a high eating and cooking quality exhibits lower amylose content, protein content, and starch gelatinization temperature and higher gel consistency and pasting viscosity [7,8,9]. The previous study by this research group demonstrated that rice eating and cooking quality was significantly positively correlated with the starch peak and breakdown viscosity and the starch peak intensity, while it was significantly negatively correlated with the starch final viscosity and setback viscosity and the starch retrogradation enthalpy and degree [10]. However, the relationship between rice eating quality and the starch fine structure is complex. Zhu et al., (2021) [7] found that the japonica rice cultivars Daohuaxiang2 and Nangeng46 both achieved high eating quality; however, Daohuaxiang2 exhibited longer amylopectin chain lengths, higher amylose content, thicker starch lamellae, and larger starch particles, while Nangeng46 produced shorter amylopectin chain lengths, lower amylose content, thinner starch lamellae, and smaller starch particles.

Nitrogen (N) is a fundamental element for rice production as well as a prerequisite and necessary measure for increasing grain yield. However, large amounts of N fertilizer are applied during rice production to achieve high yield, and the excessive use of N fertilizer results in the deterioration of grain eating quality due to the increase in protein content and decrease in gel consistency. Moreover, excessive N fertilization causes decreased N use efficiency and environmental problems, such as soil acidification, nitrous oxide emission, and water pollution. Inefficient N application is particularly common for *japonica* rice in the lower reaches of the Yangtze River in China, where the average N application rate is 300 kg ha^−1^, which is about 67% higher than China’s average and about twice as high as the global average [11]. Optimized cultivation strategies are necessary to compensate for the negative effects of the reduced N application rate on rice grain yield. Plant density is as important as N application, and it is a critical agronomic measure affecting grain yield and quality in rice [12,13], maize [14], and wheat [15] cropping systems. Previous studies have reported that reduced N application with dense planting (RNDP) can serve as an optimized cultivation method for compensating for the yield loss and improving the N use efficiency of crops with fewer environmental costs [16,17,18,19]. For example, Chong et al., (2023) suggested that reducing the N input caused a significant yield loss in rice, while increasing the planting density partially compensated for the yield loss, and a decrease in the grain protein content was observed when both N input reduction and planting density increase were implemented [17]. Fu et al., (2021) suggested that an N rate reduced by 23% with a planting density increased by 50% enhanced grain yield by 23.9–29.9% in the double rice cropping system [20]. But Wei et al., (2021) found that an N rate reduced by 15% with a planting density increased by 20% had a comparable yield for *japonica* inbred rice and a decreased yield for *indica* hybrid rice in a rice–wheat cropping system [21]. Altogether, research has shown that the planting density and N application rate are the two most important crop management practices influencing the crop grain yield and quality.

Many previous researchers have studied the effects of simultaneous changes in the N application rate and planting density on the grain yield and N use efficiency for rice. A previous study by this research group also found that dense planting with a reduced N rate could significantly increase the N partial factor productivity under a one-time fertilization of controlled-release urea [22]. However, less information has been reported regarding the effect of RNDP on rice eating quality and starch structure and properties. Therefore, a two-year field experiment was conducted to investigate the rice grain yield, eating quality and starch structure, and properties under RNDP in a rice–wheat cropping system. The specific objectives of this case study were to (1) determine whether RNDP can improve rice yield and grain eating quality simultaneously; and (2) investigate the effect of RNDP on rice starch structure and properties. The results of this case study can provide technical support for the simultaneous improvement of yield and quality in large-scale rice production.

## 2. Materials and Methods

### 2.1. Experimental Site and Growth Conditions

**Figure 1:**
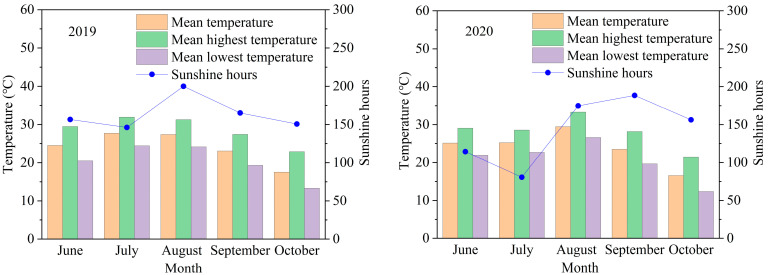
Seasonal average temperature, highest temperature, lowest temperature and sunshine hours in 2019 and 2020.

The field experiments were performed in 2019 and 2020 at an experimental site in Xinghua Town, Jiangsu Province, China (33°05′ N, 119°58′ E). The soil type in the experimental farm was loamy clay with 26.7 g kg^−1^ organic matter, 1.87 g kg^−1^ total N, 13.40 mg kg^−1^ Olsen-P, and 150.60 mg kg^−1^ available potassium (K). The seasonal average temperature and sunshine hours were measured in 2019 and 2020 (Figure 1).

### 2.2. Experimental Material and Design

Nangeng 9108, a soft and inbred *japonica* rice variety, was used in this study. Rice seeds were sown in plastic seedling trays on 26 May 2019 and 22 May 2020. Twenty-five-day old seedlings were transplanted on 20 June 2019 and 16 June 2020. According to the local large-scale rice cultivation practices, the conventional N (CN) rate of 300 kg ha^−1^ and a normal planting density 25.65 × 10^4^ ha^−1^ were set as the control. The two reduced N treatments utilized in this study consisted of 20% reduced N (RN) with a normal planting density and RN with dense planting (RNDP, with a ~20% increase in the planting density). Phosphorus (P) and K were applied as basal fertilizer in the three N treatments at fertilization rates of 90 kg ha^−1^ P and 90 kg ha^−1^ K, respectively. The experimental plot area was 30 m^2^ with three replications. The plots were separated by a soil ridge with plastic film. Water management comprised flooding at the tillering stage, midseason drainage, reflooding, and moist intermittent irrigation after heading. Rice diseases, insects, and weeds were controlled using chemicals.

### 2.3. Sampling and Measures

#### 2.3.1. Grain Yield and Its Components

The grain yield was determined by harvesting 50 hills from a 10 m^2^ area in the center of each plot at maturity. The panicle number of 50 consecutive hills was counted to examine the panicle number per hectare. The number of spikelets per panicle was also determined, and the filled and unfilled spikelets were counted to calculate the grain filled percentage. One thousand full grains were weighed three times to determine the average 1000-grain weight.

#### 2.3.2. Amylose Content, Protein Content, and Gel Consistency

The amylose content of rice flour was quantified using the iodine binding method. KjelecTM 8400 equipment (FOSS, Copenhagen, Denmark) was utilized for the determination of the N content in rice flour, and the protein content was calculated by multiplying the N content by a factor of 5.95. The gel consistency was determined according to the rice gel extension method. 

#### 2.3.3. Taste and Cooking Properties

The taste quality of cooked rice was assessed using an STA-1A rice sensory analyzer (Satake, Osaka, Japan). In brief, 30 g of milled rice was rinsed in a stainless-steel container and then transferred into a 50 mL aluminum box containing 40 mL of water. The milled rice was cooked in a rice cooker (Z06YA3-S2, Supor, Hangzhou, China). Following the cooking process, the sensory properties (including the palatability, hardness, and stickiness) of the cooked rice were evaluated using a rice sensory analyzer.

#### 2.3.4. Flour and Starch Isolation

The rice grains were initially subjected to a polishing process, which was followed by grinding into flour using a mill (FOSS 1093 Cyclotec Sample Mill, Tecator, Hoganas, Sweden) equipped with a 0.5 mm screen. Subsequently, starch was extracted from the rice grains.

#### 2.3.5. X-ray Diffraction Analysis of Starch

The XRD patterns were obtained using an X-ray diffractometer (D8 Advance, Bruker, Germany). Starch samples were analyzed at 200 mA and 40 kV with a scanning range of 3–35°(2θ) and a scanning speed of 0.02°. The relative crystallinity (%) was determined by calculating the ratio of the crystalline area to the total area using MDI Jade 6.5 software (Materials Data, Inc., Livermore, CA, USA).

#### 2.3.6. Small Angle X-ray Scattering Analysis of Starch

The lamellar structure of starch samples was determined using a Bruker NanoStar SAXS instrument equipped with a Vantec 2000 detector (Bruker NanoStar, Vantec 2000, Bruker, Germany) and pin-hole collimation for point focus geometry. The SAXS parameters were analyzed utilizing the simple graphical method.

#### 2.3.7. Attenuated Total Reflectance-Fourier Transform Infrared Analysis of Starch

ATR-FTIR spectroscopy was performed to determine the starch structural order using a Varian 7000 FTIR spectrometer (Agilent Technologies, Santa Clara, CA, USA). Prior to deconvolution, the spectra were baseline-corrected in the range of 1200 to 800 cm^−1^ using Resolutions Pro V.5.0.0 software (Agilent Technologies, Santa Clara, CA, USA). The absorbance values at 1045 and 1022 cm^−1^ were obtained from the deconvoluted spectra.

#### 2.3.8. Gelatinization Properties

The starch gelatinization properties were evaluated using a differential scanning calorimetry (DSC) instrument (200-F3, Netzsch, Selb, Germany).

#### 2.3.9. Pasting Properties

The pasting properties of rice flour were assessed using a rapid visco-analyzer manufactured by Techmaster, Newport Scientific (Sydney, Australia).

### 2.4. Statistical Analysis

The data were analyzed using Microsoft Excel 2016 and SPSS 17.0 (SPSS, Chicago, IL, USA). The figures were generated using Origin 8.5 (Origin Lab, Hampton, MA, USA). The analysis of variance and mean comparison were performed based on the least significant difference test at a significance level of *p* < 0.05.

## 3. Results

### 3.1. Grain Yield

**Figure 2:**
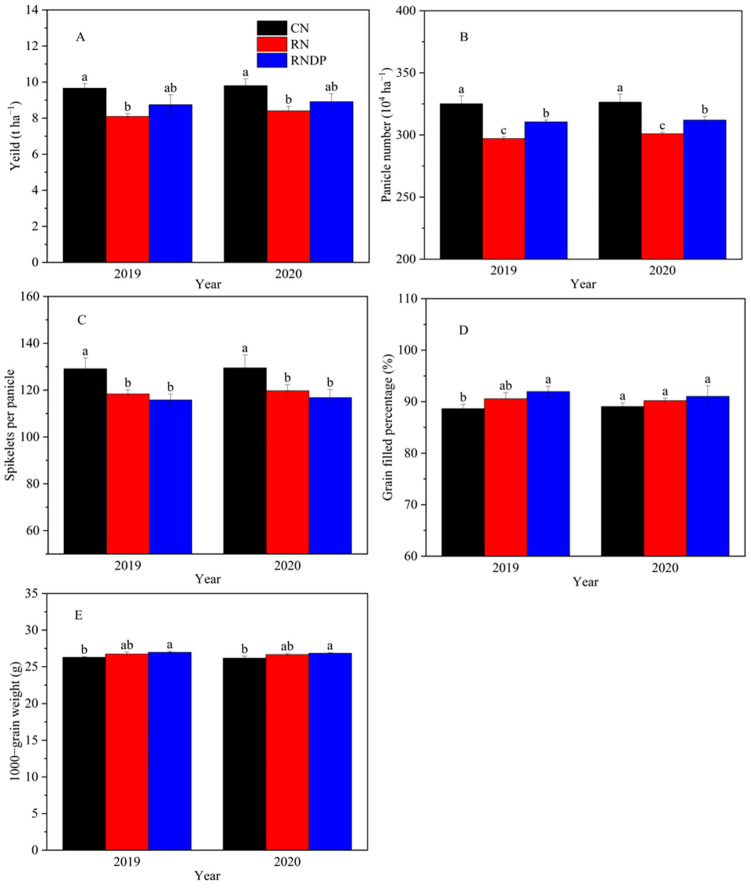
Effect of reduced nitrogen application with dense planting on rice yield (**A**) and yield components (**B**–**E**). CN, conventional nitrogen rate; RN, reduced nitrogen rate; RNDP, reduced nitrogen rate with dense planting. Lowercase letters in the figure indicate significant differences (*p* < 0.05).

As shown in Figure 2A, RN and RNDP treatments both decreased the grain yield compared with the CN treatment in the two years of the study. Compared to the CN treatment, the RN treatment significantly decreased grain yield by 16.24% and 14.27% in 2019 and 2020, respectively. The RNDP treatment increased grain yield by 8.06% and 6.02% compared with the RN treatment in 2019 and 2020, respectively. There was no significant difference in grain yield between the CN and RNDP treatments in both years. Further analysis of the yield components (Figure 2B–E) showed that compared with the CN treatment, the RN and RNDP treatments significantly decreased the panicle number and number of spikelets per panicle but increased the grain filled percentage and grain weight. The RNDP treatment significantly increased the panicle number compared with the RN treatment, and there was no significant difference in the number of spikelets per panicle, the grain filled percentage, and the grain weight between the RNDP and RN treatments.

### 3.2. Grain Eating Quality

The N application rate significantly influenced the palatability parameters of cooked rice (Table 1). Compared with the CN treatment, both reduced N treatments (RN and RNDP) markedly enhanced the appearance, stickiness, balance and taste value of cooked rice in the two years, while the hardness of cooked rice significantly decreased. In addition, there were no significant differences in the palatability parameters of cooked rice between the RN and RNDP treatments.

### 3.3. Amylose Content, Protein Content, and Gel Consistency

The amylose content, protein content and gel consistency serve as the evaluation index of rice eating quality. Compared with the CN treatment, both the RN and RNDP treatments exhibited lower protein content, and enhanced gel consistency and amylose content in the two years (Table 2). In particular, the RN treatment significantly reduced the protein content and markedly increased the gel consistency. The gel consistency was significantly higher in the RNDP treatment than that in the CN treatment in 2019.

### 3.4. Starch Crystal Structure

**Figure 3:**
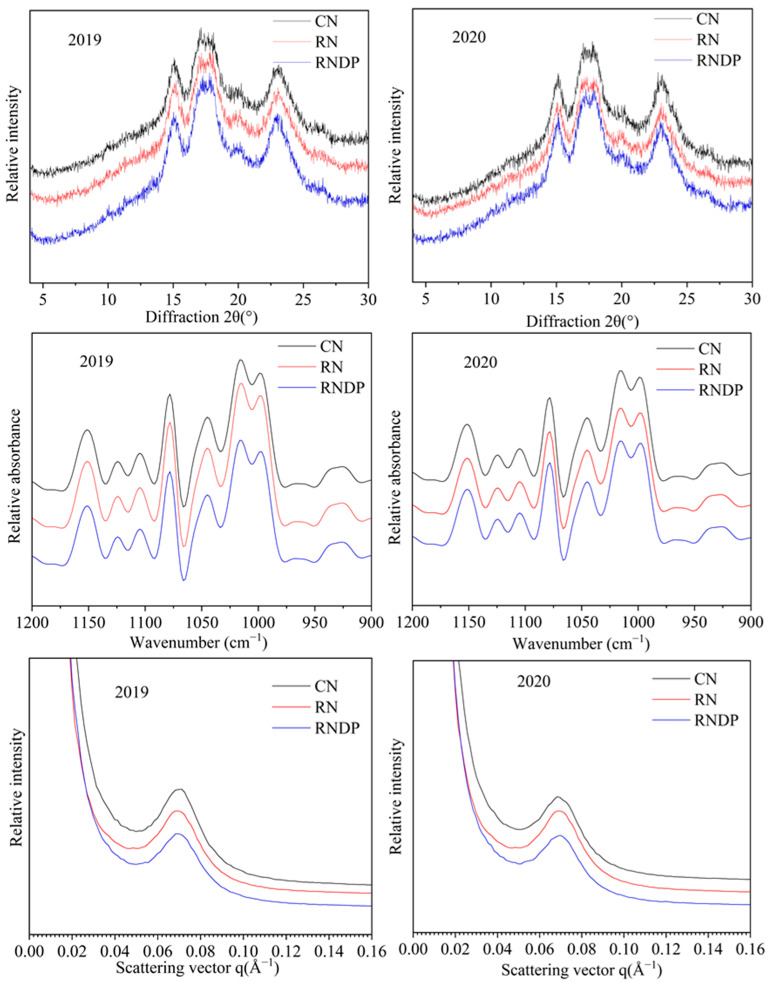
Effect of reduced nitrogen with dense planting on XRD patterns, ATR-FTIR, and SAXS of rice starch. CN, conventional nitrogen rate; RN, reduced nitrogen rate; RNDP, reduced nitrogen rate with dense planting; XRD, X-ray diffraction; ATR-FTIR, attenuated total reflectance-Fourier transform infrared; SAXS, small angle X-ray scattering.

The X-ray powder diffraction patterns of rice starch grown under reduced N conditions are displayed in Figure 3. Rice starch obtained from different N treatments displayed A-type diffraction patterns. The relative crystallinity calculated based on the XRD patterns ranged from 18.90% to 21.35% (Table 3). Compared to the CN treatment, the RN treatment significantly decreased the degree of crystallinity in both years. The RNDP treatment also exhibited lower relative crystallinity compared to the CN treatment, but the difference was not significant.

The deconvoluted ATR-FTIR starch spectra were examined to determine the short-range degree of order (Figure 3). The ratio of bands at 1045 and 1022 cm^−1^ reflected the short-range degree of ordered structures in starch. The 1045/1022 cm^−1^ ratios of the rice starch obtained from the three different N treatments are presented in Table 3. Compared to the CN treatment, the reduced N treatments (RN and RNDP) exhibited higher ratios of 1045/1022 cm^−1^, but there was no significant difference among the different N treatments.

The lamellar structures of rice starches under different N treatments were investigated using SAXS (Figure 3). The lamellar intensity (*I*_max_) and lamellar distance (*D*) in rice starches are summarized in Table 3. The values of *I*_max_ and *D* ranged from 190.97 to 238.56 and from 9.03 to 9.18, respectively. Compared to the CN treatment, both reduced N treatments (RN and RNDP) markedly decreased the lamellar intensity; however, there was no significant difference among the CN, RN, and RNDP treatments.

### 3.5. Thermal Properties of Starch

The thermal properties of rice starch under different N treatments were analyzed using differential scanning calorimetry (Table 4). The onset (*To*), peak (*Tp*), and conclusion (*Tc*) gelatinization temperatures showed comparable values among the CN, RN and RNDP treatments. However, the RN treatment showed a downward trend in gelatinization temperatures compared with the CN treatment. The gelatinization enthalpy (Δ*H*gel), retrogradation enthalpy (Δ*H*ret), and retrogradation degree (%R) differed significantly in different N treatments. Compared to the CN treatment, both the RN and RNDP treatments decreased the gelatinization enthalpy, retrogradation enthalpy and retrogradation degree.

### 3.6. Pasting Properties of Rice Flour

The rapid visco-analyzer was employed to obtain the pasting properties of rice flour exposed to different N treatments, which reflected the gelatinization, disintegration, swelling and gelling properties of starch. Compared to the CN treatment, both the RN and RNDP treatments increased the peak viscosity, trough viscosity, final viscosity, and breakdown, while the setback was decreased (Table 5). However, the RNDP treatment exhibited comparable value in terms of the pasting properties compared with the RN treatment.

### 3.7. Correlation between Grain Eating Quality and Starch Properties

**Figure 4:**
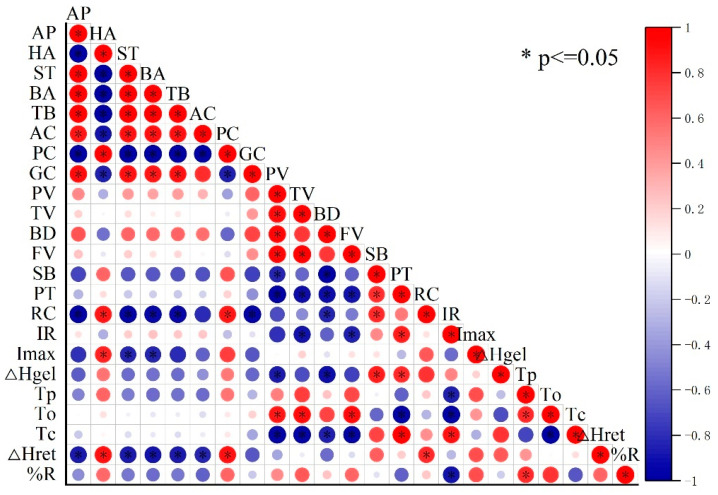
Correlation analysis of grain eating quality indexes and starch properties. AP, appearance; HA, hardness; ST, stickiness; BA, balance; TB, taste value; AC, amylose content; PC, protein content; GC, gel consistency; PV, peak viscosity; TV, trough viscosity; BD, breakdown; FV, final viscosity; SB, setback; PT, pasting temperature; RC, relative crystallinity; IR, IR ratio of 1045/1022 cm^−1^; *To*, onset temperature; *Tp*, peak temperature; *Tc*, conclusion temperature; Δ*H*gel, gelatinization enthalpy; Δ*H*ret, retrogradation enthalpy; %R = Δ*H*ret/Δ*H*gel × 100.

The grain eating quality was highly significantly correlated with the starch properties (Figure 4). The taste value, which serves as the core index of grain eating quality, was significantly positively correlated with the appearance, stickiness, and balance after rice grain cooking. In contrast, the taste value was significantly negatively correlated with the protein content and hardness. In addition, the starch relative crystallinity and retrogradation enthalpy were significantly negatively correlated with the taste value. Moreover, a negative correlation was detected between the taste value and starch gelatinization temperature and the enthalpy and *I*_max_.

## 4. Discussion

### 4.1. Effects of Reduced N Rate with Dense Planting on Rice Grain Yield

Considering the decline in N use efficiency and the rise in environmental pollution resulting from excessive N application during crop production, reduced N application provides a direct and effective approach to addressing these issues albeit with potential impacts on crop grain yield. Numerous studies have demonstrated that reduced N application leads to a decrease in the number of panicles and grains per panicle, as well as the overall accumulation of dry matter [17,23,24], while increasing the planting density of rice can enhance the grain yield by improving the number of panicles and the total dry matter accumulation for crop [17,20]. Hence, RNDP has been recommended to maintain or increase crop grain yield while improving N use efficiency. Previous studies have found that RNDP treatments result in higher or comparable grain yield compared to normal N application rates, thus causing a reduction in yield, while significantly increasing N use efficiency in rice. The differences between previously reported results are due to variations in the reduced N rates and increased planting densities in addition to the application of reduced N rates during different fertilization periods. Fu et al., (2021) found that reducing the N rate by 23% with the planting density increased by 50% enhanced grain yield by 23.9–29.9% in a double rice cropping system due to a 26.1–37.4% increase in the number of panicles [20]. Wei et al., (2021) found that reducing the N application rate by 15% with a 20% increase in planting density (RNDP) resulted in a comparable yield for japonica inbred rice and a decreased yield for indica hybrid rice in a rice–wheat cropping system [21]; this was caused by increases in the panicle numbers and grain filled percentage coupled with decreases in the number of spikelets per panicle and the total spikelets. Chen et al., (2021) demonstrated that reducing N application by 25% at the basal and tillering stages of rice combined with 20% and 40% higher planting densities maintained grain yield for early rice and late rice [16]. In this study, compared to the conventional N application rate, reducing the N rate by 20% in the basal, tillering, and panicle stages significantly decreased the panicle numbers and spikelets per panicle, ultimately reducing the rice grain yield by 16.24% and 14.27% in 2019 and 2020, respectively. However, the combination of reduced N with an increase of ~20% in the planting density enhanced the number of panicles and thus compensated for the partial loss yield under the above reduced N application (Figure 2). Therefore, the synthesis of previous research and the present study suggests that a moderate reduction in the basal or tillering N application rate coupled with an increase in the planting density can increase the number of panicles and maintain high and stable and high rice grain yield. However, the beneficial effects of long-term N reduction on soil fertility require further verification.

### 4.2. Effects of Reduced N Rate with Dense Planting on Rice Eating Quality and Starch Structure and Properties

N input affects rice grain quality, especially the eating and cooking quality of rice, which is closely associated with the composition of starch and protein in rice grains. Previous studies have demonstrated that increasing the N application rate enhances the grain protein content, which is significantly negatively correlated with the rice eating quality [25,26]. In this study, reducing the N rate by 20% (the RN treatment) significantly decreased the protein content compared to the CN treatment. The RNDP treatment reduced the protein content compared to the CN treatment but increased the protein content compared to the RN treatment. However, Chong et al. (2023) reported that reducing the N input or increasing the planting density both resulted in decreased protein content, and decreasing the N input had a stronger impact on protein content than increasing the planting density [17]. The results of the present study showed that increasing the planting density improved the protein content. Except for protein content in rice grain, the composition, structure, and functional properties of starch also play crucial roles in determining the eating and cooking quality of rice. In general, rice with a high taste quality rice exhibits lower amylose content and gel consistency, higher stickiness after cooking and breakdown viscosity, and a lower starch gelatinization temperature [6,27,28]. Previous studies suggest that increasing N application alters the structure and properties of rice starch, leading to the deterioration of the rice eating quality and starch functional properties [29,30,31]. With the increase in the N application rate, the amylose content decreased [32], the starch relative crystallinity increased [33,34], and the starch pasting temperature and gelatinization temperature and enthalpy were higher [9,34]. In the present study, compared to the CN treatment, the RN and RNDP treatments increased the amylose content, but the difference did not reach a significant level. The relative crystallinity and lamellar intensity of starch were lower in the RN and RNDP treatments than those in the CN treatment, resulting in the lower starch gelatinization enthalpy, retrogradation enthalpy, and retrogradation degree found in the RN and RNDP treatments. The correlation analysis also revealed a significant negative relationship between the taste value and the starch relative crystallinity, lamellar intensity, and starch retrogradation enthalpy. These results are consistent with previous reports that the decreased relative crystallinity of starch, as well as the reduced gelatinization temperature and enthalpy of starch, contribute to high taste quality [29,33,34]. The pasting properties also reflect the grain eating quality of rice. Rice with good eating quality has a higher breakdown viscosity and lower setback viscosity. Zhu et al., (2017) [35] and Huang et al., (2020) [6] suggested that increasing N application decreased the pasting properties of rice, which resulted in lower peak and breakdown viscosity and higher setback viscosity. The results of the present study showed that the breakdown of starch was higher in the RN and RNDP treatments than that in the CN treatment with the opposite result for setback viscosity. Altogether, the results demonstrated that the RNDP treatment produced high eating quality in rice, which was attributed to the lower protein content, the decreased relative crystallinity, lamellar intensity, gelatinization enthalpy, and retrogradation enthalpy of starch, the higher gel consistency, and the enhanced starch breakdown viscosity.

## 5. Conclusions

In this study, compared to the CN treatment, the RNDP treatment achieved a comparable grain yield and high eating quality. The high eating quality achieved under the RNDP treatment was attributed to the increases in the gel consistency, pasting viscosity, and stickiness after cooking in addition to the decreased protein content. Further analysis of the starch structure and properties revealed that the RNDP treatment reduced the relative crystallinity and lamellar intensity of starch as well as the starch gelatinization enthalpy and retrogradation enthalpy.

## Figures and Tables

**Table 1:** Effect of reduced nitrogen with dense planting on rice cooking and eating quality.

Year	Treatment	Appearance	Hardness	Stickiness	Balance	Taste Value
2019	CN	6.55 ± 0.05 b	6.55 ± 0.05 a	6.80 ± 0.00 b	6.55 ± 0.05 b	69.85 ± 0.45 b
	RN	7.50 ± 0.10 a	6.10 ± 0.00 b	7.60 ± 0.30 a	7.50 ± 0.10 a	75.70 ± 0.90 a
	RNDP	7.30 ± 0.10 a	6.20 ± 0.20 b	7.50 ± 0.30 a	7.30 ± 0.00 a	74.45 ± 0.05 a
2020	CN	6.70 ± 0.40 b	6.45 ± 0.25 a	6.95 ± 0.35 b	6.75 ± 0.45 b	70.95 ± 2.65 b
	RN	7.30 ± 0.00 a	6.15 ± 0.05 a	7.55 ± 0.05 a	7.40 ± 0.00 a	75.05 ± 0.15 a
	RNDP	7.20 ± 0.10 a	6.15 ± 0.05 a	7.40 ± 0.10 a	7.30 ± 0.10 a	74.20 ± 0.60 a

CN, conventional nitrogen rate; RN, reduced nitrogen rate; RNDP, reduced nitrogen rate with dense planting. The values are presented as means ± SD. Lowercase letters within the same column indicate significant differences (*p* < 0.05).

**Table 2:** Effect of reduced nitrogen with dense planting on amylose content, protein content and gel consistency of rice.

Year	Treatment	Amylose Content (%)	Protein Content (%)	Gel Consistency (mm)
2019	CN	11.35 ± 0.05 a	8.23 ± 0.18 a	86.55 ± 0.15 b
	RN	12.08 ± 0.19 a	7.49 ± 0.17 b	92.15 ± 1.45 a
	RNDP	11.69 ± 0.14 a	7.71 ± 0.09 ab	91.35 ± 1.15 a
2020	CN	11.33 ± 0.08 a	8.21 ± 0.05 a	87.65 ± 4.85 b
	RN	12.18 ± 0.27 a	7.45 ± 0.10 b	90.60 ± 0.10 a
	RNDP	11.76 ± 0.20 a	7.68 ± 0.65 ab	88.50 ± 2.10 ab

CN, conventional nitrogen rate; RN, reduced nitrogen rate; RNDP, reduced nitrogen rate with dense planting. The values are presented as means ± SD. Lowercase letters within the same column indicate significant differences (*p* < 0.05).

**Table 3:** Effect of reduced nitrogen with dense planting on the relative crystallinity, IR ratio and SAXS parameters of rice starch.

Year	Treatment	Relative Crystallinity (%)	IR Ratio of 1045/1022 cm^−1^	SAXS Parameters	
				*I_max_* (Counts)	D (nm)
2019	CN	21.15 ± 0.92 a	0.702 ± 0.01 a	238.56 ± 7.50 a	9.03 ± 1.41 a
	RN	18.90 ± 0.28 b	0.710 ± 0.01 a	204.39 ± 8.68 b	9.08 ± 0.92 a
	RNDP	19.20 ± 0.71 ab	0.711 ± 0.00 a	190.97 ± 5.34 b	9.09 ± 0.75 a
2020	CN	21.35 ± 0.78 a	0.741 ± 0.02 a	209.27 ± 15.17 a	9.18 ± 0.44 a
	RN	19.75 ± 0.92 b	0.751 ± 0.01 a	193.48 ± 12.11 b	9.06 ± 0.65 a
	RNDP	20.00 ± 1.70 ab	0.758 ± 0.01 a	191.23 ± 6.45 b	9.12 ± 0.42 a

CN, conventional nitrogen rate; RN, reduced nitrogen rate; RNDP, reduced nitrogen rate with dense planting; SAXS, small angle X-ray scattering. The values are presented as means ± SD. Lowercase letters within the same column indicate significant differences (*p* < 0.05).

**Table 4:** Effect of reduced nitrogen with dense planting on the thermal properties of rice starch.

Year	Treatment	*To* (°C)	*Tp* (°C)	*Tc* (°C)	Δ*H*gel (J/g)	Δ*H*ret (J/g)	%R
2019	CN	61.03 ± 0.24 a	67.14 ± 0.29 a	73.34 ± 0.44 a	10.00 ± 1.07 a	2.86 ± 0.22 a	28.63 ± 5.30 a
	RN	60.83 ± 0.33 a	66.88 ± 0.16 a	73.05 ± 0.07 a	8.71 ± 0.22 b	2.35 ± 0.21 b	26.98 ± 3.04 b
	RNDP	60.90 ± 0.14 a	67.03 ± 0.38 a	73.27 ± 1.04 a	9.17 ± 0.64 ab	2.47 ± 0.07 b	26.94 ± 1.14 b
2020	CN	59.07 ± 0.38 a	66.86 ± 0.01 a	76.94 ± 0.38 a	11.55 ± 0.42 a	3.07 ± 0.99 a	26.57 ± 9.50 a
	RN	58.98 ± 0.12 a	66.78 ± 0.12 a	76.08 ± 0.12 a	10.63 ± 0.93 ab	2.35 ± 0.20 b	22.08 ± 3.78 b
	RNDP	59.25 ± 0.35 a	66.81 ± 0.22 a	76.08 ± 0.35 a	9.61 ± 1.09 b	2.21 ± 0.93 b	23.01 ± 12.41 ab

*To*, onset temperature; *Tp*, peak temperature; *Tc*, conclusion temperature; Δ*H*gel, gelatinization enthalpy; Δ*H*ret, retrogradation enthalpy; %R = Δ*H*ret/Δ*H*gel × 100. CN, conventional nitrogen rate; RN, reduced nitrogen rate; RNDP, reduced nitrogen rate with dense planting. The values are presented as means ± SD. Lowercase letters within the same column indicate significant differences (*p* < 0.05).

**Table 5:** Effect of reduced nitrogen with dense planting on pasting properties of rice.

Year	Treatment	Peak Viscosity (cP)	Trough Viscosity (cP)	Breakdown (cP)	Final Viscosity (cP)	Setback (cP)	Pasting Temperature (°C)
2019	CN	2420.0 ± 50.0 b	1515.0 ± 28.0 a	905.0 ± 22.0 b	2095.0 ± 25.0 a	−325.0 ± 25.0 a	72.8 ± 0.0 a
	RN	2569.0 ± 0.0 a	1514.5 ± 29.5 a	1054.5 ± 29.5 a	2107.5 ± 17.5 a	−461.5 ± 17.5 b	72.4 ± 0.4 a
	RNDP	2555.0 ± 67.0 a	1581.5 ± 74.5 a	973.5 ± 7.5 ab	2203.5 ± 67.5 a	−351.5 ± 0.5 ab	72.8 ± 0.0 a
2020	CN	2135.0 ± 86.0 b	1364.5 ± 100.5 a	770.5 ± 14.5 b	1899.5 ± 92.5 a	−235.5 ± 6.5 a	74.0 ± 0.4 a
	RN	2292.0 ± 108.0 a	1400.5 ± 122.5 a	891.5 ± 14.5 a	1955.5 ± 126.5 a	−336.5 ± 18.5 b	73.7 ± 0.0 a
	RNDP	2246.0 ± 135.0 a	1374.0 ± 156.0 a	872.0 ± 21.0 a	1927.0 ± 169.0 a	−319.0 ± 34.0 b	73.7 ± 0.0 a

CN, conventional nitrogen rate; RN, reduced nitrogen rate; RNDP, reduced nitrogen rate with dense planting. The values are presented as means ± SD. Lowercase letters within the same column indicate significant differences (*p* < 0.05).

## Data Availability

The original contributions presented in the study are included in the article, further inquiries can be directed to the corresponding author.
